# Accelerating cancer evolution: a dark side of SIRT1 in genome maintenance

**DOI:** 10.18632/oncotarget.487

**Published:** 2012-05-09

**Authors:** WenYong Chen

**Affiliations:** Department of Cancer Biology, Beckman Research Institute, City of Hope, Duarte, CA.

One important aim in cancer treatment is to kill cancer cells to control tumor growth and achieve remission. However, cancer cells have strong capability to adapt to hostile environment and evolve under stress. This posts a major challenge to the treatment as most of current therapeutic strategies fail to address how to prevent cancer cells from evolution while trying to kill them. The outcome of such therapeutic strategies is the emergence of harder-to-treat malignancies once they relapse. This has been clearly recognized in targeted cancer therapy using tyrosine kinase inhibitors [1]. For instance, chronic myelogenous leukemia (CML) can be effectively treated with imatinib mesylate, an inhibitor of BCR-ABL tyrosine kinase that causes the disease. However, CML cells, especially those in advanced phases of the disease, can rapidly develop resistance by acquisition of genetic mutations of BCR-ABL that in turn require stronger inhibitors to treat. Acquisition of genetic mutations of the targeted oncogenes or their closely related genes widely occurs in targeted cancer therapy [1]. Understanding the mechanisms how cancer cells evolve under therapeutic stress may help devise new strategies to improve cancer treatment.

In a recent study, Wang et al showed that mammalian stress response gene *SIRT1* is a key player promoting acquisition of genetic mutations for CML drug resistance [2]. *SIRT1* encodes a protein deacetylase that deacetylates histones and non-histone proteins for diverse cellular functions including DNA damage repair. In the model system the authors used, BCR-ABL mutations are acquired through a *de novo* process that is dependent on BCR-ABL expression [3]. SIRT1 inhibition by small molecule inhibitors or gene knockdown blocks *de novo* acquisition of BCR-ABL mutations in blast crisis CML cells and prevents CML cell relapse from imatinib treatment. Two more recent reports have shown that *SIRT1* is activated by BCR-ABL in both kinase dependent and independent manners, and that SIRT1 activation promotes BCR-ABL mediated leukemogenesis and CML stem/progenitor cell survival from imatinib treatment [4, 5]. The study by Wang et al provides new insight that SIRT1 pathway may remain active after BCR-ABL kinase activity is inhibited and mediate subsequent acquisition of genetic mutations under imatinib therapeutic stress. The effect of SIRT1 is not limited to BCR-ABL and CML cells, as SIRT1 inhibition also effectively suppresses *de novo* mutation acquisition of *HPRT* (hypoxanthine phosphoribosyl transferase) gene in CML and prostate cancer cells when they are treated with the chemotherapeutic agent camptothecin. Interestingly, the authors showed that the role of SIRT1 in promoting mutation acquisition is associated with its ability to enhance aberrant DNA damage repair, in particular, the error-prone non-homologous end joining (NHEJ) repair.

The findings by Wang et al apparently contrast with previous reports that SIRT1 maintains genome integrity [6, 7], which would predict that loss of SIRT1 could increase genetic instability and mutagenesis. Intriguingly, whereas chromosomal abnormality is observed in *SIRT1*^−/−^ embryonic stem cells [6, 7], impaired DNA damage response is similarly observed in *SIRT1*^−/−^ mouse embryonic fibroblasts [7] and in SIRT1 knockdown CML cells [2]. Furthermore, SIRT1 knockdown reduces the efficiency of NHEJ and homologous recombination repair in CML cells [2] and previously, osteosarcoma cells [6]. Therefore, one plausible explanation for the difference is that SIRT1 may play a role for genome maintenance in both normal and cancer cells; but, there may be an important distinction of SIRT1 functions in normal versus cancer cells in this regard. In embryonic cells, loss of SIRT1 leads to gross chromosomal abnormality; however, cells bearing abnormal chromosomes may not survive. The impact of SIRT1 knockout on genome integrity in normal somatic cells has not been examined, but is expected less than in embryonic cells. This is supported by the fact that *SIRT1*^−/−^ mice, once survive from early developmental defect, do not exhibit the increased incidence of spontaneous tumors [4] and referenced therein. In contrast to normal cells, roles of SIRT1 in cancer cells may be more complicated. SIRT1 may help cancer genome maintenance by increasing DNA damage repair in response to the increased production of reactive oxygen species as a result of tumorigenesis. However, as the fidelity of DNA damage repair is compromised in cancer cells [8], new genetic mutations may arise as a consequence of misrepair. With the increased SIRT1 expression in cancer cells to enhance survival, genetic mutations or chromosomal lesions may be better tolerated, allowing cells to bypass apoptosis. Chemotherapy likely accentuates this process and stimulates evolution of mutant cells in response to therapeutic stress. Wang et al showed that SIRT1 may promote cancer evolution through enhancing Ku70-mediated error-prone NHEJ repair [2]. Interestingly, this process resembles adaptive mutations in bacteria induced by environmental stress, which is promoted by activation of stress response genes and error-prone DNA damage repair [9]. It is provocative to suggest that stress-induced mutagenesis may be a conserved pathway from bacteria to certain mammalian (cancer) cells to survive hostile environment, which involves cellular stress response and error-prone DNA damage repair.

Although molecular details how therapeutic stress signals mutation acquisition and cancer evolution are yet to be worked out, the findings by Wang et al have clear translational implication. Given the roles of SIRT1 in CML molecular pathobiology and drug resistance, SIRT1 inhibition in combination with BCR-ABL inhibitors may not only help eradicate residual CML stem cells but also prevent them from acquiring resistant mutations and evolution. Such a strategy may benefit treatment of other types of malignancies as well.
